# The MPO−463G>A Polymorphism and Lung Cancer Risk: A Meta-Analysis Based on 22 Case–Control Studies

**DOI:** 10.1371/journal.pone.0065778

**Published:** 2013-06-20

**Authors:** Jun-Ping Yang, Wen-Bo Wang, Xiao-Xi Yang, Lei Yang, Li Ren, Fu-Xiang Zhou, Liu Hu, Wei He, Bai-Yu Li, Yan Zhu, Huan-Gang Jiang, Yun-Feng Zhou

**Affiliations:** 1 Department of Radiotherapy Oncology, The Third Affiliated Hospital of Wuhan University, Wuhan, Hubei, People’s Republic of China; 2 Department of Chemo-Radiotherapy Oncology, Zhongnan Hospital, Wuhan University, Wuhan, Hubei, People’s Republic of China; University of Porto, Portugal

## Abstract

**Background:**

Myeloperoxidase (MPO) is an endogenous oxidant enzyme that produces reactive oxygen species (ROS) and may be involved in lung carcinogenesis. The MPO−463G>A polymorphism influences MPO transcription and has been associated with lung cancer susceptibility. However, the association between the MPO−463G>A polymorphism and lung cancer risk remains controversial.

**Method:**

To investigate the effect of this polymorphism on lung cancer susceptibility, we performed a meta-analysis based on 22 published case–control studies including 7,520 patients with lung cancer and 8,600 controls. **O**dds ratios (ORs) with 95% confidence intervals (CIs) were used to assess the strength of the association.

**Results:**

Overall, there was no evidence for significant association between MPO−463G>A polymorphism and lung cancer susceptibility (for AA versus GG: OR = 0.91, 95%CI = 0.67–1.24; for GA versus GG: OR = 0.87, 95% CI = 0.78–0.98; for AA/GA versus GG: OR = 0.90, 95% CI = 0.80–1.01; for AA versus GA/GG: OR = 0.96, 95% CI = 0.72–1.28). In the stratified analyses by ethnicity, source of controls and smoking status, we also did not find any significant association between them.

**Conclusions:**

In summary, this meta-analysis suggests MPO−463G>A polymorphism may not be a risk factor for developing lung cancer. However, further prospective well-designed population-based studies with larger sample size are expected to validate the results.

## Introduction

Lung cancer is one of the most common malignancies and the leading cause of cancer-related deaths in the United States and worldwide. It is estimated that in 2011, approximately 221,000 new cases will be diagnosed and 156,900 deaths due to lung cancer will occur in the United States [1]. The exact mechanism of lung carcinogenesis is still unclear. Lung cancer may be a multifactorial disease that resulted from complex interactions between genetic and environmental factors [2]. It has been estimated that cigarette smoking is responsible for 85–90% of lung cancer development [3]. Although smoking can account for the majority of lung cancer, most chronic smokers still do not develop lung cancer. This suggests that lung cancer susceptibility differs among individuals and might have genetic factors which may influence the risk of lung cancer among these who are exposed to tobacco smoke carcinogens.

Myeloperoxidase (MPO) as an endogenous oxidant lysosomal enzyme available in polymorphonuclear neutrophils and monocytes catalyzes the reaction between the chloride ion and hydrogen peroxide and generates hypochlorous acid and other reactive oxygen species (ROS) [4]. The reactive by-products generated by MPO can cause oxidative damage in vivo to biomolecules, such as DNA, protein and lipids, and cause cellular alterations that may lead to carcinogenesis.

The human MPO gene located on chromosome 17q23.1 consists of 12 exons and 11 introns [5] and there are at least 319 different polymorphism sites in the MPO gene (http://www.ncbi.nlm.nih.gov/projects/SNP). The most extensively studied single nucleotide polymorphism (SNP) was −463G>A polymorphism (rs2333227) located in the promoter region of the MPO gene. Since Austin et al first published the MPO−463G>A polymorphism in 1993 [5], many subsequent studies have consecutively reported the relationship between this polymorphism and different cancer types including esophagus, breast, bladder, brain and lung cancer and so forth. Among them, the relationship between lung cancer risk and the MPO−463G>A polymorphism was the most extensively studied, It has been previously suggested that there was an association between the GG+GA genotype of MPO and a decreased risk of lung cancer [6]–[10]. However, other studies have failed to confirm such the association [8], [11]–[24]. The exact relationship between MPO−463G>A polymorphism and susceptibility to lung cancer is inconclusive or conflicting. Up to now, there have been two relevant published meta-analysis studies involving the MPO−463G>A polymorphism and lung cancer risk [25], [26], among which one was published in Chinese and the other one was dealt with the meta-analysis on overall cancer susceptibilities. Unfortunately, those two meta-analyses all failed to adopt the most likely appropriate genetic model and lacked subgroups analyses such as smoking status and thus compromised the authentic values of statistical results. In addition, an increasing number of new studies between MPO−463G>A polymorphism and lung cancer risk are available.

So it is necessary and significant to perform a meta-analysis to explore the association between the MPO−463G>A polymorphism and lung cancer risk. Therefore, we performed a meta-analysis on all eligible case–control studies to estimate the overall lung cancer risk of MPO−463G>A polymorphism and to quantify heterogeneity among the individual eligible studies.

## Methods

### Search Strategy

Eligible literatures published before the end of September 2012 were identified by the search of PUBMED, EMBASE, ISI Web of Science databases using combinations of the following keywords: “Myeloperoxidase”, “MPO”, “polymorphism” or “variant” and “lung cancer” or “lung carcinoma” without restriction on language. All relevant publications were reviewed. Articles in reference lists were also hand-searched for potentially relevant publications.

### Inclusion and Exclusion Criteria

Studies included had to meet all the following inclusion criteria: (a) the diagnosis of lung cancer patients were confirmed histologically or pathologically; (b) a case–control study on MPO−463G>A polymorphism and lung cancer; (c) sufficient available data for estimating an odds ratios (ORs) with 95% confidence intervals (CIs). Major reasons for exclusion of studies were as follows: (i) not for lung cancer study, (ii) only case population and (iii) duplicate of previous publication.

### Data Extraction

Two investigators (JP Yang, B Wang) extracted the data independently including first author, year of publication, country, ethnicity (Caucasian, Asian and Mixed), source of controls (hospital-based studies, population-based studies), genotyping methods, matching variables, number of genotypes in cases and controls. Discrepancies were adjudicated by the third investigator (YF Zhou) until consensus was achieved on every item. Because two studies [7], [11] only provided the information of genotypes as “GG” and “AA/GA” without data for all three genotypes, we could only calculate the odds ratios (ORs) for the AA/GA versus GG model. We also abstracted the information of smoking status from available studies, and cigarette smoking status was strategically classified as never smokers, light smokers and heavy smokers.

### Statistical Analysis

For control group of each study, the allelic frequency was calculated and the observed genotype frequencies of the MPO−463G>A polymorphism were assessed for Hardy-Weinberg equilibrium (HWE) by using the chi-square test. We calculated the strength of the association between MPO−463G>A polymorphism and lung cancer risk by odds ratios (ORs) corresponding to 95% confidence intervals (CIs). The pooled ORs were performed for homozygote model (GA versus GG), heterozygote model (AA versus GG), dominant model (AA/GA versus GG) and recessive model (AA versus GA/GG) respectively. Stratified analyses were also performed by ethnicity, source of controls and smoking status respectively. Heterogeneity was evaluated with a chi-square-based Q test (*P*<0.10 was considered significant) [27]. When heterogeneity was present, the random effects model was used to calculate the pooled ORs, whereas the fixed effects model was used [28]. Galbraith plot was used to determine the main sources of the heterogeneity [29], [30]. The one-way sensitivity analyses were performed to assess the stability of the results, namely, a single study in the meta-analysis was deleted each time to reﬂect the inﬂuence of the individual data set to the pooled ORs [31]. The publication bias of literatures was assessed using funnel plot and funnel plot asymmetry was assessed by the method of Egger’s linear regression test [32]–[34]. The significance of the intercept was determined by the t test suggested by Egger and *P*<0.05 was considered significant. All statistical analyses were performed with STATA software (version 12.0; STATA Corporation, College Station, TX), and all tests were two sided.

## Results

### Study Characteristics

**Figure 1:**
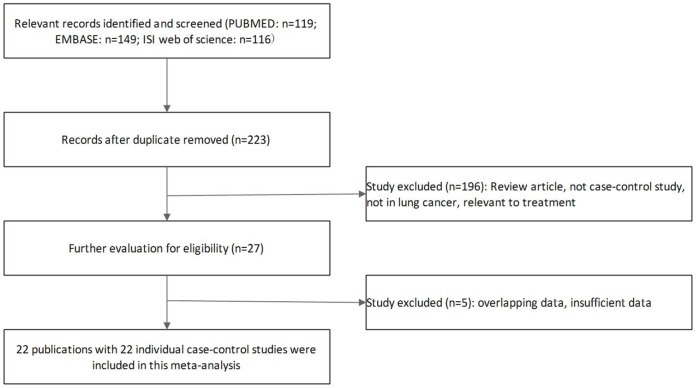
Flow chart of study selection based on the inclusion and exclusion criteria.

For lung cancer susceptibility related to MPO−463G>A polymorphism, Study selection process was shown in **Figure 1**. 22 case–control studies met the inclusion criteria including7,520 patients with lung cancer and 8,600 controls in this meta-analysis[6]–[24], [35]–[37]. The characteristics of included studies and distribution of the frequencies of MPO−463G>A polymorphism on lung cancer were summarized in **Table 1** and **Table 2** respectively. Overall, there were 9 studies of Caucasians, 6 studies of Asians, 7 of mixed population, and 11 studies of population-based, 11 studies of hospital-based. Six studies [8], [15], [16], [18], [22], [23] collected the information on possible confounding factors like smoking status. All cases were pathologically confirmed and Almost controls were mainly matched for age and sex. Most polymorphisms in the control subjects were in Hardy–Weinberg equilibrium.

**Table 1:** Main characteristics of studies included in the meta-analysis.

First author	Year	Country	Ethnicity	Control source	Genotyping method	Matching
Arslan	2011	Turkey	Caucasian	PB	PCR–RFLP	NA
Klinchid	2009	Thailand	Asian	HB	PCR–RFLP	NA
Yoon	2008	Korea	Asian	PB	Taqman	age
Zienolddiny	2008	Norway	Caucasian	PB	Taqman	age and sex
Yang	2007	Korea	Asian	HB	Taqman	NA
Larsen	2006	Australia	Caucasian	HB	PCR–RFLP	age
Park	2006	Korea	Asian	HB	PCR–RFLP	age and sex
Chan	2005	China	Asian	HB	PCR–RFLP	age and sex
Skuladottir	2005	Denmark	Caucasian	PB	PCR–RFLP	age and sex
Harms	2004	USA	Mixed	PB	PCR–RFLP	NA
Liu	2004	USA	Mixed	PB	PCR–RFLP	NA
Chevrier	2003	France	Caucasian	HB	Taqman	age
Dally, H	2002	Germany	Caucasian	HB	PCR–RFLP	sex
Feyler	2002	France	Caucasian	HB	PCR–RFLP	age and sex
Kantarci	2002	USA	Mixed	HB	PCR–RFLP	sex and ethnicity
Lu	2002	China	Asian	PB	PCR–RFLP	age and sex
Schabath	2002	USA	Mixed	HB	PCR–RFLP	age,sex and ethnicity
Xu	2002	USA	Mixed	PB	PCR–RFLP	NA
Misra	2001	Finland	Caucasian	PB	Taqman	age
Cascorbi	2000	Germany	Caucasian	HB	PCR–RFLP	age and sex
Marchand	2000	USA	Mixed	PB	PCR–RFLP	age,sex and ethnicity
London	1997	USA	Mixed	PB	PCR–RFLP	age,sex and ethnicity

*Abbreviations* HB: Hospital-based studies; PB: Population-based studies; PCR-RFLP: Polymerase chain reaction–restriction fragment length polymorphism; NA: Not available.

**Table 2:** Distribution of MPO−463G>A polymorphism among lung cancer cases and controls included in the meta-analysis.

First author	Year	Sample size		cases			Controls			*P* for HWE
		Cases	Controls	GG	GA	AA	GG	GA	AA	
Arslan	2011	106	271	67	35	4	136	110	21	0.85
Klinchid	2009	88	81	59	29^ a^		57	24^ a^		NA
Yoon	2008	213	213	180	31	2	175	35	3	0.42
Zienolddiny	2008	258	297	150	74	34	179	109	9	0.11
Yang	2007	318	353	269	49	0	283	68	2	0.33
Larsen	2006	627	624	382	205	40	383	210	31	0.75
Park	2006	432	432	353	76	3	356	72	4	0.87
Chan	2005	75	162	44	28	3	118	42	2	0.41
Skuladottir	2005	122	396	75	47^ a^		270	126^ a^		NA
Harms	2004	110	119	56	47	7	59	56	4	0.03
Liu	2004	830	1119	490	296	44	692	386	41	0.15
Chevrier	2003	243	245	135	98	10	140	93	12	0.49
Dally, H	2002	625	340	429	173	23	218	105	17	0.35
Feyler	2002	150	172	98	42	10	96	63	13	0.55
Kantarci	2002	307	307	192	106	9	181	111	15	0.70
Lu	2002	314	320	248	60	6	227	87	6	0.48
Schabath	2002	375	378	235	126	14	202	157	19	0.10
Xu	2002	989	1128	599	343	47	697	390	41	0.13
Misra	2001	315	311	191	108	16	206	84	21	<0.01
Cascorbi	2000	196	196	141	49	6	117	75	4	0.04
Marchand	2000	323	437	234	77	12	294	116	27	<0.01
London	1997	339	703	353	136	16	401	243	59	0.01

*Abbreviations* HWE: Hardy–Weinberg Equilibrium; NA: not applicable; a: number of GA+AA.

### Meta-analysis Results

**Figure 2:**
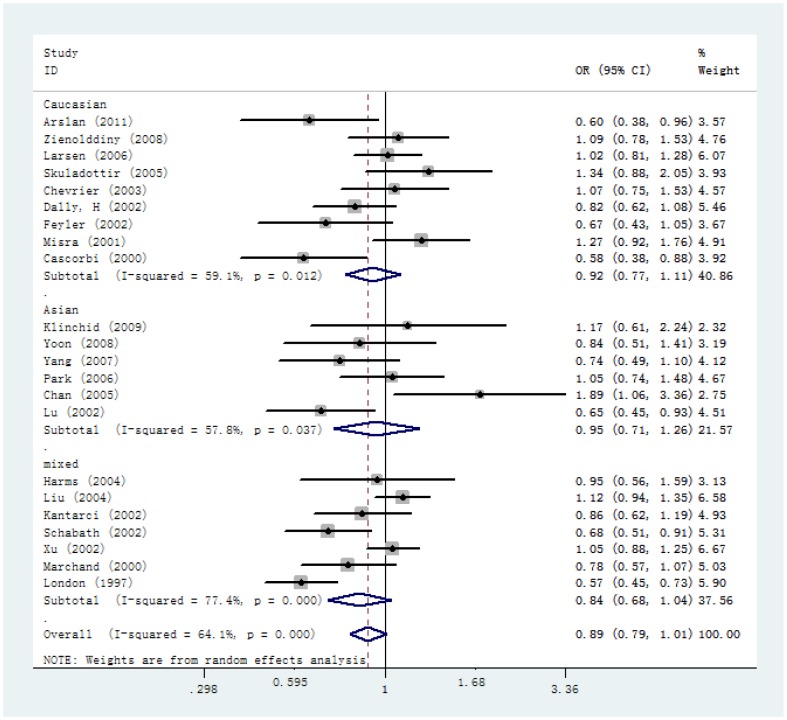
Forest plots of lung cancer risk associated with the MPO−463G>A polymorphism for AA/GA versus GG model in the stratified analyses by ethnicities.

**Table 3** listed the results of the association between the MPO−463G>A polymorphism and lung cancer risk. Overall, there was no evidence for significant association between MPO−463G>A polymorphism and lung cancer susceptibility (for AA versus GG: OR = 0.91, 95%CI = 0.67–1.24; for AA/GA versus GG: OR = 0.90, 95%CI = 0.80–1.01; for AA versus GA/GG: OR = 0.96, 95%CI = 0.72–1.28) (**Figure 2**). In the stratified analysis by ethnicity, source of controls and smoking status, we also did not find any significant association between MPO−463G>A polymorphism and lung cancer risk. MPO −463G>A polymorphism was a low protective susceptibility gene in lung cancer development in homozygote model (for GA versus GG: OR = 0.87, 95%CI = 0.78–0.98), however, when stratified by ethnicity, source of controls and smoking status, we also did not find any significant association between them (for Caucasian population: OR = 0.86, 95%CI = 0.71–1.05, for Asian population: OR = 0.92, 95%CI = 0.67–1.25, for mixed population: OR = 0.86, 95%CI = 0.73–1.02, for population-based studies: OR = 0.87, 95%CI = 0.74–1.03, for hospital-based studies: OR = 0.87, 95%CI = 0.74–1.02, for never smokers: OR = 0.77, 95%CI = 0.43–1.38, for light smokers: OR = 1.00, 95%CI = 0.79–1.27, for heavy smokers: OR = 0.92, 95% CI = 0.69–1.23).

**Table 3:** Meta-analyses of the MPO −463G>A polymorphism on lung cancer risk.

Subgroup	Number	AA versus GG(heterozygote)		GA versus GG(homozygote)		AA/GA versus GG (dominant)		AA versus GA/GG (recessive)	
	N/Cases/Controls	OR (95% CI)	*P_value_/P_het_*	OR (95% CI)	*P_value_/P_het_*	OR (95% CI)	*P_value_/P_het_*	OR (95% CI)	*P_value_/P_het_*
**Total**	22/7520/8600	0.91(0.67,1.24)	0.56/<0.01	0.87(0.78,0.98)	0.02/<0.01	0.90(0.80,1.01)	0.07/<0.01	0.96(0.72,1.28)	0.77/<0.01
**Ethnicity**									
**Caucasian**	9/2642/2848	1.03(0.64,1.67)	0.89/<0.01	0.86(0.71,1.05)	0.13/0.02	0.92(0.77,1.11)	0.38/0.01	1.09(0.67,1.76)	0.74/<0.01
**Asian**	6/1440/1561	0.92(0.46,1.85)^a^	0.82/0.46	0.92(0.67,1.25)	0.59/0.04	0.95(0.71,1.26)	0.70/0.04	0.95(0.47,1.90)^a^	0.87/0.56
**Mixed**	7/3439/4191	0.79(0.47,1.33)	0.38/<0.01	0.86(0.73,1.02)	0.09/0.01	0.84(0.68,1.04)	0.11/<0.01	0.84(0.53,1.33)	0.46/<0.01
**Study design**									
**PB**	11/4085/5310	0.96(0.58,1.60)	0.87/<0.01	0.87(0.74,1.03)	0.10/<0.01	0.91(0.76,1.09)	0.28/<0.01	1.00(0.62,1.64)	0.99/<0.01
**HB**	11/3436/3290	0.88(0.67,1.14)^a^	0.32/0.46	0.87(0.74,1.02)	0.09/0.03	0.88(0.75,1.03)	0.11/0.03	0.92(0.71,1.20)^a^	0.54/0.58
**Smoking status**									
**Never smokers**	2/174/709	1.07(0.23,5.00)	0.93/NA	0.77(0.43,1.38)	0.38/NA	0.79(0.54,1.14)	0.21/NA	1.17(0.25,5.40)	0.84/NA
**Light smokers**	6/825/943	1.05(0.60,1.84)^a^	0.86/0.83	1.00(0.79,1.27)^a^	0.98/0.37	0.92(0.74,1.13)^a^	0.41/0.18	1.07(0.62,1.86)^a^	0.81/0.84
**Heavy smokers**	5/744/688	1.39(0.75,2.60)^a^	0.30/0.85	0.92(0.69,1.23)^a^	0.57/0.60	0.82(0.66,1.03)^a^	0.09/0.21	1.58(0.86,2.90)^a^	0.15/0.47

*Abbreviations* N:Number of studies; NA: Not applicable; *P_het_* : Probability of heterogeneity; a: Fixed-effects model was used when ***P_het_***≥0.1, otherwise, random model was used.

### Test of Heterogeneity

**Figure 3:**
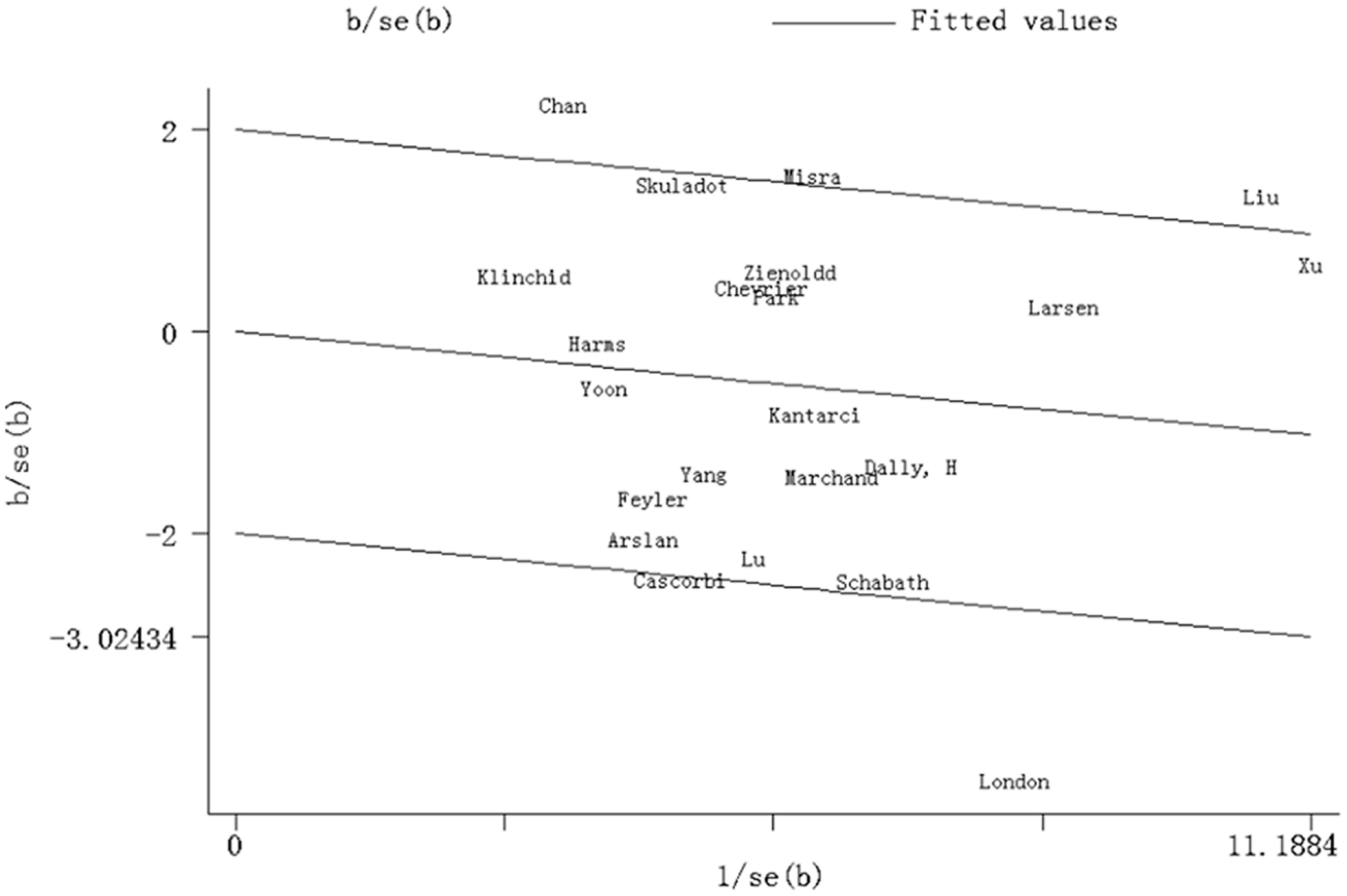
Galbraith plot analysis of the amount of heterogeneity from all the included studies (AA/GA versus GG). The Y-axis shows the ratio of the log OR to its standard error (SE), and the x-axis shows the reciprocal of the SE. At a 2 standard deviation distance parallel to the regression line, the 2 lines create an interval. Studies lacking in heterogeneity would lie within the 95% confidence interval.

Significant heterogeneity existed in four genetic models of the MPO−463G>A polymorphism (AA versus GG, GA versus GG, AA/GA versus GG, AA versus GA/GG) (**Table 3**). Galbraith plot analyses of all included studies were used to assess the potential sources of heterogeneity. Chan, Liu and London’s studies [10], [35] were found to be the main contributors of heterogeneity in the AA/GA versus GG model (**Figure 3**). The significance of pooled ORs with 95%CIs in the AA/GA versus GG model in both overall comparison and subgroup analyses was not influenced by omitting those three studies (Data not shown).

### Sensitivity Analysis

**Figure 4:**
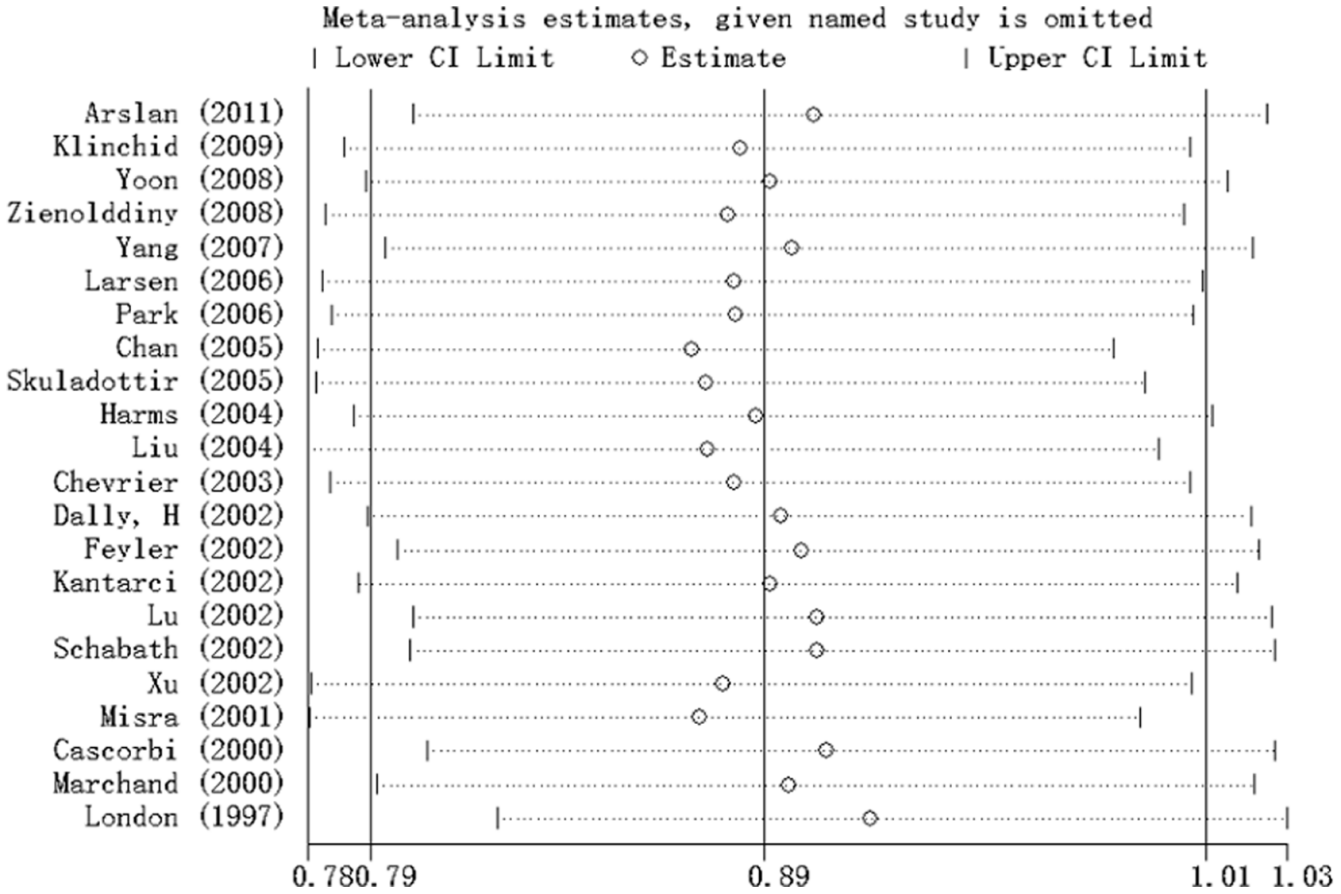
Sensitivity analysis conducted to assess the influence of each study on the pooled ORs by individual studies omission in AA/GA versus GG model.

Sensitivity analysis was performed through sequentially excluding individual studies. Statistically similar results were obtained after sequentially excluding each study in GA/AA versus GG model (**Figure 4**) and the corresponding pooled ORs in the other genetic models were not materially altered (Data not shown), suggesting stability and liability of this meta-analysis.

### Publication Bias

**Figure 5:**
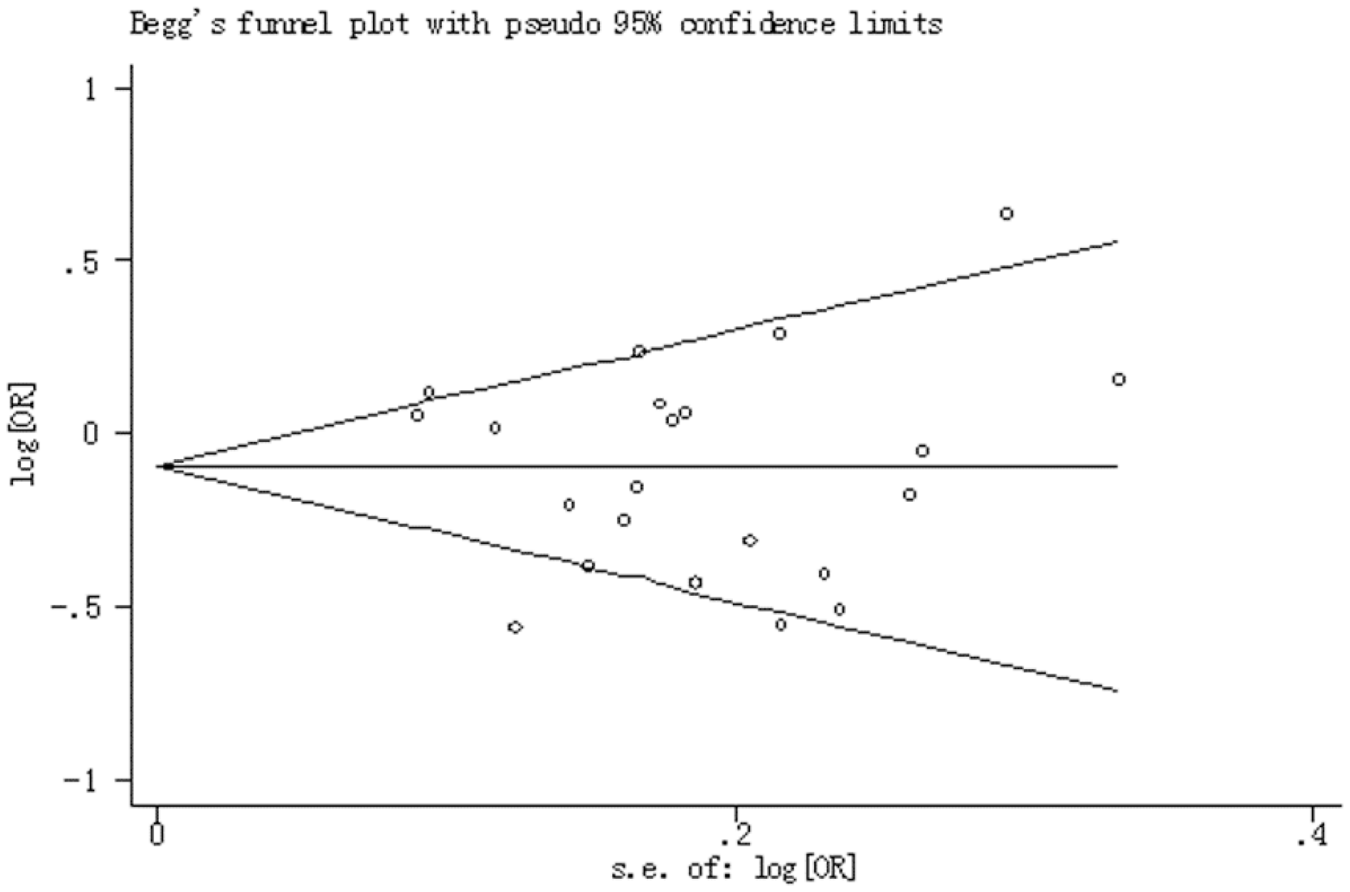
Begg’s funnel plot between the MPO−463G>A polymorphism and lung cancer risk in AA/GA versus GG model.

The funnel plot and Egger’s test did not provide any obvious evidence of publication bias that examined the MPO−463G>A polymorphism and lung cancer risk. The shape of funnel plots did not reveal any evidence of funnel plot asymmetry (**Figure 5**), Egger's test further provided statistical evidence of funnel plot symmetry for AA versus GG (*P* = 0.55), GA versus GG (*P* = 0.27), AA/GA versus GG (*P* = 0.56) and for AA versus GA/GG (*P* = 0.61).

## Discussion

As we all know, lung cancer is a complex multifactorial and multistage process, in which both host genetic factors and environmental factors are involved [2]. Individual genetic susceptibility has been suggested to correlate with lung cancer risk. In the current meta-analysis, on the basis of 22 case-control studies providing the information on the MPO−463G>A polymorphism and lung cancer involving 7,520 cases and 8,600 controls, we did not find any significant association between the MPO−463G>A polymorphism and lung cancer risk in any genetic model and also the similar results stratified by ethnicity, source of controls and smoking status respectively. The one-way sensitivity analyses suggested the stability and liability of the results in this meta-analysis. Publication bias was not observed in this study. Our meta-analysis suggests that the MPO −463G>A polymorphism is not associated with lung cancer development.

Oxidative stress occurs when the excessive production of reactive oxygen species (ROS) overwhelms the antioxidant defense system. Increasing evidences suggests variability in these genes involved in oxidative stress may determine the level of oxidative stress in the organism and play a crucial role in carcinogenesis [4], [5]. Therefore, it is rational to speculate that certain genetic variants or polymorphisms in the genes involved in oxidative stress may have an impact on cancer risk. MPO is an endogenous oxidant enzyme that generates reactive oxygen species (ROS) that may be involved in carcinogenesis [4], [38]. MPO−463G>A polymorphism was located in the promoter region of the MPO gene. The G allele acts as a strong stimulatory protein 1 (SP1) transcription factors binding site, which reacts with SP1 to elevate MPO transcriptional activity [39]. Therefore the guanosine (G) to adenosine (A) nucleotide base substitution is associated with disruption of the SP1 binding site and thus reduces 25 times MPO gene expression and decreases the enzyme levels. So it is biologically plausible that MPO−463G>A polymorphism may modulate the risk of lung cancer. Therefore, many studies have investigated the role of MPO−463G>A polymorphism in the pathogenesis of lung cancer [6]–[24], [35]–[37]. However, the results remain conﬂicting rather than conclusive. There are several possible explanations for this discordance, such as small sample size, ethnic background, uncorrected multiple hypothesis testing, and publication bias. Meta-analysis is a statistical procedure for combining the results of several studies to produce a single estimate of the major effect with enhanced precision. So we performed a meta-analysis on 22 eligible case–control studies to estimate the overall lung cancer risk of MPO−463G>A polymorphism, whereas no significant associations were found between them in any genetic model in this meta-analysis.

Population stratification is an area of concern that can lead to spurious evidence for the association between a marker and cancer and suggest a possible role for ethnic differences in genetic backgrounds.

Previous studies have found a wide variation in the A allele frequency of the MPO−463G>A polymorphism across different ethnicities. The −463A allele frequency was 22.8% in European population, but approximately 14.7% in Asian population [40]. When stratified according to ethnicity in this meta-analysis study, no significant associations were found in any of the genetic models in the Caucasian, Asian and mixed population. Although hospital-based studies may have inherent selection biases, we also did not find any positive result in the stratified analyses by population-based and hospital-based studies. These results suggested that the different ethnicities and source of controls did not influence the association between the MPO−463G>A polymorphism and lung cancer risk.

Lung cancer have been characterized as causally related to cigarette smoking [41]. MPO transforms tobacco smoke procarcinogens, such as benzo(α)pyrene and arylamines, into highly carcinogenic intermediates, such as benzo(α)pyrene dio-epoxide. Since MPO−463G>A polymorphism may be associated with weaker transcriptional activity and decreases the enzyme levels, carcinogens contained in cigarette smoking will not be metabolically activated by MPO enzyme, therefore this polymorphism has been suggested to have a protective effect against the development of cancers related to smoking such as lung cancer [42], [43]. Whereas in our meta-analysis study, the MPO−463G>A polymorphism was shown to have no statistically significant protective effect in light smokers and heavy smokers. The exact mechanism for this inverse result was not clear. That may be due to the limited number of study subjects related to smoking which may have low power of statistical test, so further large-scale researches between MPO−463G>A polymorphism and risk of lung cancer in smokers are expected to confirm the results.

Several limitations of this meta-analysis should be addressed. First, when interpreting the results of this meta-analysis, heterogeneity was a potential problem and the origins of heterogeneity may include many factors, such as the differences in control characteristics and diverse genotyping methods; Second, the lack of detailed information such as age, sex and lifestyle of the patients in some studies, limited further stratification, and more accurate ORs would be corrected for age, sex and other factors that were associated with lung cancer risk. Nevertheless, our meta-analysis has some advantages. First, the well-designed search and selection method significantly increased the statistical power of this meta-analysis. Second, we could perform a subgroup analysis to address a possible interaction between smoking parameters and MPO−463G>A polymorphism. Third, the results did not show any evidence of publication bias.

In summary, our meta-analysis suggests that the MPO −463G>A polymorphism may not be a risk factor for lung cancer. However, lung cancer may be a multifactorial disease that resulted from complex interactions between genetic and environmental factors, we could not collect the detailed original data of MPO gene polymorphisms and then we were not able to investigate potential gene-gene, gene- environment interactions. Further prospective researches using adjusted individual data with large sample studies which study the relationship between MPO−463G>A polymorphism and the risk of lung cancer are necessary and expected, which would lead a better, comprehensive understanding of the association between MPO−463G>A polymorphism and lung cancer risk.

## Supporting Information

Checklist S1 MOOSE Checklist.
(DOC)
