# Supplementary material for: The MPO−463G>A Polymorphism and Lung Cancer Risk: A Meta-Analysis Based on 22 Case–Control Studies
Source: PLoS One. 2013 Jun 20;8(6):e65778. doi: 10.1371/journal.pone.0065778 (PMC3688689; doi:10.1371/journal.pone.0065778)
Supplement: Checklist S1 — MOOSE Checklist. (DOC) [file pone.0065778.s001.doc]

**MOOSE Checklist**

| **Criteria** | | **Brief description of how the criteria were handled in the meta-analysis** |
| --- | --- | --- |
| **Reporting of background should include** | |  |
| √ | Problem definition | Myeloperoxidase (MPO) is an endogenous oxidant enzyme that produces reactive oxygen species (ROS) and may be involved in lung carcinogenesis. The MPO−463G>A polymorphism influences MPO transcription and has been associated with lung cancer susceptibility. However, the association between the MPO−463G>A polymorphism and lung cancer remains controversial.. The potential public health impact of MPO−463G>A polymorphism on lung cancer risk remains to be summarized quantitatively. |
| √ | Hypothesis statement | MPO−463G>A polymorphism might influence the risk of lung cancer. |
| √ | Description of study outcomes | Lung cancer |
| √ | Type of exposure or intervention used | GA or GG of MPO |
| √ | Type of study designs used | We included case-control studies, cross-sectional studies. |
| √ | Study population | We placed no restriction. |
| **Reporting of search strategy should include** | |  |
| √ | Qualifications of searchers | The credentials of the two investigators Yang and Wang are indicated in the author list. |
| √ | Search strategy, including time period included in the synthesis and keywords | PubMed from 1965 –September 2012  EMBASE from 1974 –September 2012  ISI Web of Science from 1965 –September 2012  MPO, Single nucleotide polymorphism, lung cancer, Meta-analysis |
| √ | Databases and registries searched | PubMed, EMBASE and ISI Web of Science databases |
| √ | Search software used, name and version, including special features | We did not employ any search software. EndNote was used to merge retrieved citations and eliminate duplications |
| √ | Use of hand searching | We hand-searched bibliographies of retrieved papers for additional references, |
| √ | List of citations located and those excluded, including justifications | Details of the literature search process are outlined in the flow chart. The citation list is available upon request |
| √ | Method of addressing articles published in languages other than English | We placed no restrictions on language; local scientists fluent in the original language of the article were contacted for translation |
| √ | Method of handling abstracts and unpublished studies | No unpublished studies were observed. |
| √ | Description of any contact with authors | We contacted authors who had conducted studies with MPO−463G>A polymorphism and lung cancer risk. |
| **Reporting of methods should include** | |  |
| √ | Description of relevance or appropriateness of studies assembled for assessing the hypothesis to be tested | Detailed inclusion and exclusion criteria were described in the methods section. |
| √ | Rationale for the selection and coding of data | Data extracted from each of the studies were relevant to the population characteristics, study design, exposure,, and possible effect modifiers of the association. |
| √ | Assessment of confounding | No restricted for the analysis. Conducted sensitivity analyses by eliminating each study. |
| √ | Assessment of study quality, including blinding of quality assessors; stratification or regression on possible predictors of study results | The results of sensitivity analyses were very stable. |
| √ | Assessment of heterogeneity | Heterogeneity of the studies were explored within two types of study designs using Cochrane’s Q test of heterogeneity and I^2^ statistic that provides the relative amount of variance of the summary effect due to the between-study heterogeneity. |
| √ | Description of statistical methods in sufficient detail to be replicated | Description of methods of meta-analyses, sensitivity analyses and assessment of publication bias are detailed in the methods. |
| √ | Provision of appropriate tables and graphics | We included the terms used for database search, 3 summary table, 1 flow chart, 1 forest plot of all studies, 1 galbraith plot, 1 sensitivity analysis plot, 1 funnel plots to examine publish bias. |
| **Reporting of results should include** | |  |
| √ | Graph summarizing individual study estimates and overall estimate | Figure 2 |
| √ | Table giving descriptive information for each study included | Table 1 and Table 2 |
| √ | Results of sensitivity testing | Figure 4 |
| √ | Indication of statistical uncertainty of findings | 95% confidence intervals were presented with all summary estimates, *P* values and results of sensitivity analyses |
| **Reporting of discussion should include** | |  |
| √ | Quantitative assessment of bias | Sensitivity analyses indicate this non-significant association was stable. |
| √ | Justification for exclusion | We excluded studies that had used different exposure or outcome assessment for the comparison groups, or no control group. |
| √ | Assessment of quality of included studies | We discussed the results of the sensitivity analyses. |
| **Reporting of conclusions should include** | |  |
| √ | Consideration of alternative explanations for observed results | We discussed that potential unmeasured confounders such as other life style factors may have caused significant results. |
| √ | Generalization of the conclusions | No significant association was detected between MPO−463G>A polymorphism and lung cancer. |
| √ | Guidelines for future research | We recommend further prospective well-designed population-based studies with larger sample size are expected to validate those results on the associations between MPO−463G>A polymorphism and lung cancer. |
| √ | Disclosure of funding source | No funding supported this study. |
